# A qualitative study on conveyance decision-making during emergency call outs to people with dementia: the HOMEWARD project

**DOI:** 10.1186/s12873-020-0306-6

**Published:** 2020-01-29

**Authors:** Sarah Voss, Janet Brandling, Katherine Pollard, Hazel Taylor, Sarah Black, Marina Buswell, Richard Cheston, Sarah Cullum, Theresa Foster, Kim Kirby, Larissa Prothero, Sarah Purdy, Chris Solway, Jonathan Benger

**Affiliations:** 1 Faculty of Health and Applied Sciences, University of the West of England, Glenside Campus, Blackberry Hill, Bristol, BS16 1DD UK; 2 Research Design Service, University Hospitals Bristol NHS Foundation Trust, Bristol, UK; 3 Research and Audit Department, South Western Ambulance Service NHS Foundation Trust, Taunton, UK; 4 Centre for Research in Primary and Community Care, University of Hertfordshire, Hatfield, UK; 5 Faculty of Medical and Health Sciences, University of Auckland, Auckland, New Zealand; 6 Research Support Services, East of England Ambulance Service NHS Trust, Bury St. Edmunds, UK; 7 School of Social and Community Medicine, University of Bristol, Bristol, UK; 8 Research Network, Alzheimer’s Society, London, UK

**Keywords:** Prehospital care, Emergency ambulance systems, Dementia, Geriatrics, Admission avoidance

## Abstract

**Background:**

Paramedics are increasingly required to make complex decisions as to whether they should convey a patient to hospital or manage their condition at the scene. Dementia can be a significant barrier to the assessment process. However, to our knowledge no research has specifically examined the process of decision-making by paramedics in relation to people with dementia. This qualitative study was designed to investigate the factors influencing the decision-making process during Emergency Medical Services (EMS) calls to older people with dementia who did not require immediate clinical treatment.

**Methods:**

This qualitative study used a combination of observation, interview and document analysis to investigate the factors influencing the decision-making process during EMS calls to older people with dementia. A researcher worked alongside paramedics in the capacity of observer and recruited eligible patients to participate in case studies. Data were collected from observation notes of decision-making during the incident, patient care records and post incident interviews with participants, and analysed thematically.

**Findings:**

Four main themes emerged from the data concerning the way that paramedics make conveyance decisions when called to people with dementia: 1) Physical condition; the key factor influencing paramedics’ decision-making was the physical condition of the patient. 2) Cognitive capacity; most of the participants preferred not to remove patients with a diagnosis of dementia from surroundings familiar to them, unless they deemed it absolutely essential. 3) Patient circumstances; this included the patient’s medical history and the support available to them. 4) Professional influences; participants also drew on other perspectives, such as advice from colleagues or information from the patient’s General Practitioner, to inform their decision-making.

**Conclusion:**

The preference for avoiding unnecessary conveyance for patients with dementia, combined with difficulties in obtaining an accurate patient medical history and assessment, mean that decision-making can be particularly problematic for paramedics. Further research is needed to find reliable ways of assessing patients and accessing information to support conveyance decisions for EMS calls to people with dementia.

## Introduction

It is estimated that around 850,000 people in England are living with dementia [1]. Dementia is a progressive and irreversible condition resulting in a decline of cognitive, functional, behavioural and psychological abilities, and tends to be associated with a loss of independent living and social interaction [2]. An increasing number of emergency medical services (EMS) calls are to older adults, and research indicates that 14% of older adults who make an EMS call have recognised cognitive impairment consistent with dementia; the true number is possibly higher due to under-diagnosis [3].

Dementia can be a significant barrier to clinical assessment in the emergency care setting; confusion resulting from dementia may contribute to inaccuracies in the medical or medication history, and may limit an individual’s ability to comprehend questions or follow instructions [4–6]. It is often difficult to establish if confusion is due to pre-existing cognitive impairment, delirium associated with the event leading to the call, or a combination of the two [7, 8]. Nonetheless, paramedics are required to decide whether the patient can be safely treated and managed at home or in the community, or if conveyance to hospital is the most appropriate course of action. This decision may be further complicated if family members or carers express a preference for where the patient should be treated; emergency care is sometimes accessed by informal carers due to ‘desperation’ for health support [9] and unmet needs [10].

Unnecessary hospital conveyance may result in adverse outcomes for people living with dementia. Evidence suggests that people with a diagnosis of dementia are more likely to be admitted to hospital [11], and once admitted, they have poorer outcomes including: longer length of stay; higher rates of readmission; higher chance of discharge to a care home; higher mortality [12–14]. However, EMS attendances to people with dementia are commonly due to a fall [15] and there is also evidence to suggest that not conveying older adults who have fallen can lead to a high rate of subsequent emergency healthcare contacts and an increased risk of death and hospitalisation [16]. Consequently, making appropriate conveyance decisions will improve patient outcomes.

The rising demands on prehospital and emergency care are well documented, and paramedics are increasingly required to make decisions as to whether to convey a patient to hospital or manage them solely at the scene [17, 18]. Research on conveyance decisions has highlighted the complexity of decision-making for paramedics [19], and suggests that a skilled workforce is key to managing diverse patient needs and reducing unnecessary conveyance to the emergency department [20]. However, to our knowledge no research has specifically examined the process of decision-making by paramedics in relation to people living with dementia.

This qualitative study was designed to investigate the factors that influence the decision-making process of paramedics during calls to older people with dementia, with a view to providing paramedics with adequate support to enhance care in this patient group.

## Method

### Study design

This qualitative study used a combination of observation, interview and document analysis to investigate the factors influencing the decision-making process when paramedics attend calls to older people with dementia. A phenomenological approach was adopted to observe the paramedics’ decision making and explore the impact of factors such as organisation, resources and family wishes on their decisions regarding conveyance to hospital [21]. The observations were inductive and areas of interest were then explored deductively during in-depth follow up interviews with paramedics. This approach allowed authentic consideration of the way that individual participants experienced events as they occurred.

The participants in the study were paramedics and people living with dementia. A person living with dementia is the preferred terminology used to describe someone with a dementia diagnosis. In this study the people living with dementia were, by virtue of the EMS call, patients within the ambulance service. For this reason, the term ‘patient’ is used to describe the participant from the EMS perspective and person living with dementia is used in all other circumstances.

### Selection, recruitment and consent

The study was publicised to all eligible paramedics (*n* = 650) working within one region of a single United Kingdom ambulance service. Those who wished to take part were invited to contact the research team directly. Sixteen paramedics responded and were recruited from both rural and urban areas.

Patients were eligible for a case study if they:

- (Or someone on their behalf) had called an emergency ambulance;
- Had a condition that did not require immediate clinical intervention (to avoid the possibility that clinically necessary treatment would be delayed as a result of study participation);
- Were attended by a participating EMS paramedic;
- Were aged 65 years or older;
- Had an established diagnosis of dementia;
- Consented to observation of the call and analysis of the call records.

The criteria for establishing the existence of a diagnosis of dementia were:

1. Documentary evidence at the scene that the patient had a dementia diagnosis. This may have been in the form of paperwork or a care plan left by visiting care staff.
2. Verbal confirmation from the patient and/or the carer that they had been diagnosed with dementia by a General Practitioner (GP) or hospital doctor.
3. Patients already known to the ambulance service from previous calls, and identified as a person with dementia on the call record.

If no evidence of at least one of these factors was available, it was assumed that the patient did not have an established diagnosis of dementia, and they were not eligible for inclusion in the study.

### Procedure

The observational researcher (JBr) shadowed study paramedics for the duration of each shift in line with the ambulance service observer policy. Different shifts during different time periods and on different days (including nights and weekends) were observed to take account of variation in the availability of primary and community healthcare services in and out of hours. Once on scene and after initial assessment, the participating paramedic screened the patient for eligibility. If they were eligible and willing to participate, the researcher approached them to provide further information about the study and to obtain written informed consent. Capacity was assessed by paramedics as part of routine procedure; for people with dementia who were eligible but did not have capacity, the researcher determined if a personal consultee (spouse or family member) was present. In these cases, the researcher provided detailed study information and asked the consultee to advise as to what the person with dementia was likely to have decided before they lost capacity.

### Data collection

Following consent, the researcher observed and recorded all assessments and interactions. Data were collected from three sources:

1. Observation: Assessments, input from family members or others present on scene, and actions taken by the paramedic were documented as field notes.
2. Paramedic interview: The researcher asked the paramedic for clarification on decisions made and the rationale for these both during and after the call (see Appendix A for interview schedule).
3. Document analysis: The researcher took a copy of the Patient Care Record (PCR) and any referral documents that the paramedic completed during the course of the call and any subsequent handover.

Field notes and PCRs were anonymised and interviews were audio-recorded, transcribed and anonymised by the observational researcher before sharing with an independent researcher.

### Data analysis

#### First level analysis

Each of the four data sources was first analysed as a stand-alone data set. An iterative process of data reduction, constant comparison, organisation and understanding through thematic analysis was used to analyse each data source, using the method described by Braun et al. [22]. Each of these data sets was analysed by the independent researcher (KP) and checked by the observational researcher (JBr) and a second independent researcher (SV) for plausibility and validity.

#### Second level analysis

Each data source was triangulated against the others to test for similarity, contradictions and consistencies. Deviant cases were actively sought. The phenomenological approach meant that analysis primarily explored the experiences of the paramedics, focusing on the accounts they gave of their decision-making and contextual factors that influenced these decisions. This was facilitated by real-time observations, which enhanced the quality and relevance of the subsequent interviews [20]. As with the first level analysis, the triangulation and subsequent themes were checked by a second researcher.

It is important to recognise the field researcher’s characteristics [23]. She was a health researcher and previously a nurse rather than a paramedic, with experience working in the health sector and with people with dementia. This provided a sense of being a partial insider with familiarity and insight into the emergency situation which quickly enhanced the participants’ sense of ease. However, it also means the researcher entered the field with some pre-existing ideas and sympathies towards the working practices of health professionals. The length of shifts and immersion in the field gave ample opportunity for continued discussions with participants and clarification after the calls were completed. It was also important for the researcher to periodically discuss the observational episodes with the research team so that she was able to step back from the field and take a broader view of the case studies.

## Findings

Sixteen paramedics were recruited to the study, and their characteristics are shown in Table 1. The researcher observed 42 shifts over a 6 month period and attended 154 incidents. Sixty-eight of the 154 patients (41%) were aged 65 years or over. A diagnosis of dementia was established for eleven patients (13%) who were initially recruited to the observational phase of the study. Two were later excluded as further information became available during the incident that indicated they might not meet eligibility criteria. Analysis was therefore conducted on nine case studies. There were a number of additional cases where the paramedic and the researcher agreed that a patient was likely to have a cognitive impairment consistent with dementia but were unable to establish a diagnosis in accordance with the eligibility criteria. These patients were not recruited to the study.

**Table 1:** Paramedic characteristics

Participants (*n* = 16)	13 paramedic, 3 specialist paramedic			
Age (years)	Mean: 34; range: 21–57			
Gender	9 female, 7 male			
Ethnicity	16 White British			
Training level	Vocational IHCD: 3	Foundation degree: 6	BSc: 6	MSc: 1
Experience (years)	Mean 6.5; range 1–16			

This paper focuses on findings from the documentary data, observations and paramedic interviews. Four main themes emerged from the data concerning the way that paramedics make decisions during emergency calls to people with dementia: 1) Clinical condition; 2) Cognitive capacity; 3) Patient circumstances; 4) Professional influences. Each of these themes is discussed below, drawing on field notes recorded during nine emergency calls and data from interviews with nine paramedics, each of whom attended one of these events. Five patients were conveyed to hospital, two were treated at home and two did not require treatment. In one case, the paramedic provided advice and reassurance, and in the other a referral for social support was made. In the presentation of findings below, each call has been assigned a unique number (Table 2). Paramedics who participated in interviews are denoted by a research code, e.g. P1 or P2. Table 2 shows details of the ambulance calls.

**Table 2:** Details of case study calls

Calls	Setting	Gender	Carers	Reason for call	Outcome
02	Care home	Female	Care home workers	Fall/head wound	Treated at scene
03	Private residence	Male	Family carer External carers	Difficulty breathing	Reassurance
04	Private residence	Female	External carers	Feeling very unwell	Conveyed
05	Care home	Female	Care home workers	Diabetes	Conveyed
06	Care home	Female	Care home workers	Fall/fracture	Conveyed
07	Private residence	Female	Family carer External carers	Collapse	Conveyed
08	Care home	Female	Care home workers	Collapse	Conveyed
09	Care home	Male	Care home workers	Fall/head wound	Treated at scene
11	Private residence	Male	Family carer External carer	Fall	Referral for social support

### Clinical condition

The key factor influencing paramedics’ decision-making during an emergency call was the clinical condition of the patient. This was assessed through a mixture of general and clinical observation. This includes scanning the scene, surveying the patient, taking vital signs and collecting information from informants and available documentation (Table 3, quotes 1–4).

**Table 3:** Clinical condition

Quote no	Quote	Data source
1	*The paramedic and emergency care assistant started to take physical observations. They were all within normal parameters except the blood sugar which was 30 + mmols.*	Call 5, field notes
2	*We were led into a small room where four elderly women sat around eating breakfast and watching the TV … We could immediately see this was not a cardiac arrest.*	Call 8, field notes
3	*He was alert and responsive. And complaining of some pain in his lower back … from what I could gather, superficial injuries, he had a cut to his face. Complaining of this lower back pain, but it seemed that it was probably sort of lying on the floor that was causing it. He had quite a lot of mobility, so we managed to get him … off the floor … and managed to do a full assessment. Once we’d got him up, he was in no pain, he had a superficial [cut] and that was it.*	Call 11, P13 interview
4	*I think the longer we sat with him, the more reassured I was that actually, it wasn’t like a severe, life-threatening exacerbation.*	Call 3, P3 interview
5	*I think the final straw was when I did the ECG and there were some quite significant ECG changes, which I couldn’t really ignore. They didn’t necessarily imply that she was having a heart attack, or anything like that, but certainly it could’ve indicated that she had some cardiac ischaemia or some kind of electrolyte problem*	Call 4, P5 interview
6	*If she hadn’t had a high blood glucose level, and it had just been a UTI, I would’ve questioned why she was being sent to hospital … the fact that her blood glucose level was so high … I think she had undiagnosed diabetes … she did have to go in. So there wasn’t really any choice.*	Call 5, P6 interview
7	*If it’s causing you a lot of pain I think that’s a good indication that it’s not necessarily broken, but you’ve gotta suspect a fracture. Then it’s just pain relief and get them comfortable and convey.*	Call 6, P7 interview
8	*When the despatcher said it’s a broken hip … so you need an ambulance … I said to her, well I can at least go and administer pain relief.*	Call 6, P7 interview
9	*The paramedic inserted a cannula and administered IV paracetamol.*	Call 8, field notes
10	*As far as NICE head injury guidance go [*sic*], [patient]‘s on nothing that requires her to have a CT scan in hospital. She’ll be cared for, she’ll be monitored by staff. We can give them head injury advice with a view to then contact further services, either [an] NHS one or [the] treble nine services again, if [patient] deteriorates in the next seventy-two hours.*	Call 2, P2
11	*The wound was suitable to be sutured* in situ *and the paramedic was satisfied he had no further injury. So he proceeded to clean the wound, administer local anaesthetic and suture it.*	Call 9, field notes
12	*He did have a mild increased work of breathing. But he was fully alert and was interacting well. And [paramedic] had provided treatment with the nebuliser. ... Once distracted in conversation, his breathing wasn’t audible, it didn’t sound like an issue. And he walked across the room; it didn’t sound like it was troubling him.*	Call 3, P3 interview

Where the patient’s clinical condition clearly indicated that conveyance to hospital was advisable, no other factors were taken into account. The need for further assessment and any necessary treatment in the acute setting was the overriding factor in making the decision to convey to hospital (Table 3, quotes 5–9).

In two cases, paramedics provided on-scene treatment which obviated the need to convey the patient to an acute hospital setting. In another case, the patient’s clinical condition was re-assessed after on-scene treatment in order to make an appropriate decision (Table 3, quotes 10–12).

In the absence of clinical indicators enabling a clear decision about conveyance, wider factors considered by paramedics included the patient’s cognitive capacity and circumstances.

### Cognitive capacity

All the patients were older adults with a diagnosis of dementia, some of whom also exhibited signs of fear or anxiety. The degree of their cognitive capacity ranged from difficulty recalling events to difficulties with communication (Table 4, quotes 1–2).

**Table 4:** Cognitive Capacity

Quote no	Quote	Data source
1	*He had not been able to recall how the ambulance had been called. He didn’t know whether he had pressed the care line button or whether his son had called.*	Call 3, field notes
2	*The patient was voluble and seemed to be confabulating as some of his speech made sense and some did not. He did not appear to have capacity.*	Call 11, field notes
3	*Especially with a dementia patient, quite keen to stay on scene where possible. ... Where you can safety-net them, and manage any injuries they’ve sustained, in the community, where they’re comfortable. As opposed to convey them to hospital unnecessarily, and in an environment that’s unpleasant and potentially not ideally suited for dementia patients.*	Call 2, P2 interview
4	*You’re not gonna drag someone out of their house when they’ve got no signs or symptoms of a head injury for a just in case. Especially when they have got that level of dementia.*	Call 11, P13 interview
5	*Obviously, because she’s got dementia it wasn’t much of a history. And you weren’t sure how much was true and how much wasn’t. A hundred per cent [decision to convey], especially when I realised she’d got dementia. ... She’s got dementia, she’s got hip pain.. ... It’s easy to be caught out because you don’t really have the whole expertise to fully assess somebody with dementia.*	Call 6, P7 interview
6	*To be honest I think regardless of any co-morbidities that she had, she’d’ve been going in, dementia or otherwise. If she was young, old, any health problems, with those observations and that presenting condition and complaint, she’s going to hospital.*	Call 7; P8 interview
7	*I asked her what she wanted to do, and she very clearly said that she didn’t wanna be on her own and she wanted to go to hospital. Which you do sometimes get with people and that ... still doesn’t necessarily meant they need to go to hospital, so we won’t make that decision.*	Call 4, P5 interview
8	*Dragging him out of somewhere that he knows and putting him in somewhere that [he] doesn’t, is gonna cause him a lot of problems. Even if that was what the wife was hoping for.*	Call 11, P13 interview

It was apparent that most of the paramedic participants preferred not to remove patients who were living with dementia from surroundings familiar to them, unless they deemed it absolutely essential. (Table 4, quotes 3–4). However, one participant indicated that a diagnosis of dementia made it more likely that she would convey patients, particularly if communication proved difficult and she was unable to assess the patient (Table 4, quote 5). Conversely, where conveyance was definitely indicated, a diagnosis of dementia did not influence the decision made (Table 4, quote 6).

In cases where a patient or carer had a preference for conveyance to hospital, paramedics asserted that they would not convey unless they felt in was in the patient’s best interest (Table 4, quotes 7–8).

### Patient circumstances

Patient circumstances included the patient’s medical history and the support available to them at home. Information concerning these issues was gleaned from patients themselves where possible and also from carers, friends or relatives present at scene. One of the paramedics commented on how useful it could be to talk to someone who knows the patient well to find out what is usual for them (Table 5, quotes 1–2).

**Table 5:** Patient Circumstances

Quote no	Quote	Data source
1	*When I go to them I’ll always say, ‘what caused you to end up on the floor?’ And sometimes even if they have got dementia, they have got some recollection of what happened.*	Call 6, P7 interview
2	*I was able to ask [the neighbour] questions about the patient in terms of how is she compared to normal. And that’s really useful to have. ‘Cos she looked fairly pale to me, but her neighbour said she didn’t look like abnormally pale, which was reassuring.*	Call 4, P5 interview
3	*She then began to read a hospital discharge letter and notes folder. There were two folders, one appeared to be for carers, the other appeared to be more hospital related letters and previous paramedic forms.*	Call 3, field notes
4	*The paramedic read through the [care] notes and found an extensive history of mental health problems including schizophrenia, bi-polar disorder and dementia.*	Call 5, field notes
5	*Often I’ve said to care agencies ... all you need is a sheet at the front that just says a simple medical history and some contact details ... at the very least, a list of medications, allergies. ...It’s not hard to put that at the front of a care plan.*	Call 4, P5 interview
6	*The paramedic found the GP notes very difficult to read as they were erratic notes on a scrap of paper and the hand writing difficult to read.*	Call 5, field notes
7	*I definitely wasn’t thinking ‘oh, he has dementia, he has to go into hospital’; or ‘I’m not taking him to hospital, because he has dementia’. [Dementia] doesn’t play that much of a part in my decision-making. … I think it maybe just makes him a little bit more vulnerable. If you’re treating him in the community ....you’d maybe just want to make sure that there is support in place – which I think there is.*	Call 3, P3 interview
8	*If she hadn’t had dementia I probably wouldn’t’ve even mentioned not going to hospital, to be honest. But anyone in a care home immediately has a higher level of care. They’re not by themselves. They’ve not got a carer popping in like four times a day. They’ve got permanent care. So ... the ability to leave them there is higher. And with someone with dementia ... you know their condition’s going to get worse when they’re in hospital. It always does. So if you can avoid it, it’s best. But in this case it’s not really possible.*	Call 8, P10 interview
9	*He almost needs somebody kind of ... like a relative or a friend, doesn’t he, just to socially be with.*	Call 3, P3 interview

If patients were in their normal place of residence, paramedics could often access written information left by carers. However, the information was sometimes of poor quality, difficult to read or extensive; paramedics indicated that a concise summary of information would be more useful to them (Table 5, quotes 3–6).

Support seemed to be an important issue, particularly where people with dementia were living in their own homes. One factor appearing to influence participants’ decisions was the type and extent of care available to the patient in the community. For some participants, this included social support (Table 5, quotes 7–9) and the wishes of family members or informal carers (Table 4, quote 8).

### Professional influences

The data showed that paramedics also draw on specialist and other colleagues’ perspectives in order to inform their decision-making; for instance, making a call to a GP or a specialist EMS advisor. This was particularly the case when it was uncertain whether a patient needed to be conveyed to hospital. During interviews, a few participants spoke about the importance of experience and training when making these decisions, and of possible difficulties arising from being a practitioner working alone (Table 6, quotes 1–4).

**Table 6:** Professional influences

Quote no	Quote	Data source
1	*The paramedic and OT (Occupational Therapist) soon ascertained that he was uninjured, with a slight graze to his chin and an ache in his back … [They] decided to make a referral to the reablement team and dementia care.*	Call 11, field notes
2	*[The paramedic already present on the scene] had been unsure whether to admit and had spoken to a specialist paramedic for advice. As [the patient] was over 65 years the specialist was unsure about prescribing antibiotics. There was also some concern about whether this was COPD or asthma …*. *[The attending paramedic] said she was thinking it might not be of benefit to admit again but that she would like to speak to the GP. She called the GP surgery … a GP called back and they had a long conversation.*	Call 3, field notes
3	*A large element is learning on the job, past experience and that sort of thing … obviously you do your mentored practice as a trainee. That certainly helps. You progress through your course, then your mentor should be giving you more and more freedom, to the point where at the end of your course you are working independently, really.*	Call 7, P8 interview
4	*I think it’s always like tricky when you’re a lone paramedic, and you’re in a car. And you do feel a little bit more isolated. And you’ve got nobody really to consult with the decision-making. Like you’ve not got a crew-mate to bounce ideas off.*	Call 3, P3 interview
5	*When I saw that he’s had probably two hospital admissions already in March, and it’s a weekday and his GP surgery is open, and available to discuss his case with, I think I started to become a bit more keen about trying to keep him out of hospital.*	Call 3, P3 interview
6	*The paramedic discussed decision-making afterwards. She feels that they lack a good awareness of the legal implications for their decisions; and that paramedics protect their professional registration at all costs. This means that sometimes they make very cautious decisions. However, they all know people who have had to go to a coroner’s court or a tribunal by the HCPC (Health and Care Professions Council) and no one wants to be in this position*.	Call 8, field notes

Other issues informing decision-making processes which paramedic participants raised during interview included the time of day, fear of litigation and the relationship between guidelines and practitioners’ opinions (Table 6, quotes 5–6).

## Discussion

The findings indicate that the clinical condition of patients with a diagnosis of dementia is the primary reason for conveying to an acute care setting following an EMS call. The conveyance decision was also influenced by the patient’s cognitive capacity, their personal and social circumstances and other professional influences, such as the availability of information from a GP or the opinion of a colleague. This, to some extent, reflects findings from other research concerning a more general patient population. A recent review by Ebben et al. [24] found that factors influencing a non-conveyance decision are related to the professional’s competence and experience, the patient’s health status and best interest, the healthcare system and the availability of decision support.

However, the focus of this study was decision-making for EMS calls to older people with dementia. The findings indicated that where the decision was not clear-cut, a diagnosis of dementia was likely to discourage conveyance. Paramedics expressed concern about removing people unnecessarily from their own familiar surroundings, aware that this can have a detrimental effect on people living with dementia. This may create professional challenges for the paramedics making these decisions as they need to weigh up the risks to the patient associated with conveyance to hospital with the risks associated with leaving the patient at home or in the community.

Despite the apparent preference for non-conveyance, five of the nine patients in this sample were conveyed to hospital. None of these patients had a condition that required immediate treatment, and it could therefore be reasoned that difficulties in the assessment of patients resulted in the decision to convey. One paramedic discussed difficulties relating to the assessment of people with dementia, particularly when communication is an issue and it is not easy to establish the presence of injury and the level of pain that the patient is experiencing. This is an important issue as commonly used pain assessment instruments rely on self-report and the communicative capacity of the patient [25, 26]. For patients with cognitive difficulties, pain can be assessed using observational techniques [27, 28], but these observational approaches require repeat assessments over time [29] and may not be well suited to the prehospital environment. Accepted methods for assessing pain in cognitively impaired adults in prehospital care have significant limitations [30], and there is a need for further work in this area.

Research has indicated that the decision to not convey a patient is a complex one, and is often negotiated between EMS staff, the patient and the patient’s family [19, 24]. Paramedics in this study were careful to familiarise themselves with relevant information concerning the patient’s medical history and level/type of support in order to inform their decision-making. Being a participant in a study examining decision-making is likely to have made this activity more pronounced, for example describing activity that is usually unspoken. Professional factors taken into account included views from colleagues and other practitioners, as well as their own professional obligations as registered healthcare practitioners. Additional information and expertise can strengthen the rationale for a decision. Previous research has found that there can be a mismatch between policy and practice in relation to non-conveyance decisions [19]. Indeed, the findings from this study indicate that the relationship between guidelines and the paramedics’ opinions is not always harmonious, and there is a fear of disciplinary action and litigation involved in making conveyance decisions. Decision-making is an iterative process involving weighing up the risks and benefits for patients, family and the healthcare system. These factors may favour a decision to convey to hospital, particularly when there is limited access to information or alternative services, even when this is not in the patient’s best interests.

There are a number of limitations to this study which affect the transferability of the findings. Data was collected by one researcher in one division of a single ambulance service. The availability of alternatives to hospital varies widely according to location, and a larger study is needed to determine the extent to which additional services affect decision-making. In addition, the sample size for the cases was small, and it is possible that data saturation was not reached [31]. The intention was to recruit 20 patients for case studies but despite adopting a number of strategies, such as targeting particular times of day and paramedics most likely to attend calls to older people, it was not possible to recruit the target number of patients within the allocated time and budget. The paramedics and the researcher were sometimes unable to be certain whether or not a patient had a diagnosis of dementia. When there was any uncertainty, the patient was excluded and this further reduced the sample size. There is also a risk of observation bias in this study; the presence of the researcher may have influenced the paramedic decision-making and the self-selecting participant sample are not necessarily representative of the wider paramedic profession. Finally, the methodology of observing shifts in the ambulance service was resource intensive, and alternative methods should be considered for future research. However, the use of both real-time observation and subsequent interview enhanced the quality of the study in methodological terms, allowing for a comprehensive exploration of the paramedics’ decision-making processes.

## Conclusions

The study findings give rise to number of implications for research and future policy. The factors influencing conveyance decision-making for people with dementia are similar to those that guide decisions for other patient groups, and older adults in particular. However, the preference for avoiding unnecessary conveyance in people with dementia, combined with difficulties in obtaining an accurate history and assessing the condition of the patient, indicate that decision-making in this particular patient group is influenced by multiple factors and can be especially challenging. Paramedics rely heavily on information that may or may not be available or accessed on scene, such as informants, carer records and input from GPs. Further research is needed to find reliable ways of assessing patients, such as an evidence-based decision tool, and service changes are required to support access to information that can assist decision-making in people with dementia.

## Data Availability

The datasets used and/or analysed during the current study are available from the corresponding author on reasonable request.

## Abbreviations

EMS: Emergency Medical Services
GP: General Practitioner
PCR: Patient Care Record
